# The CTSA Diversity, Equity, Inclusion, and Accessibility (DEIA) Task Force’s recommendations for the CTSA program consortium

**DOI:** 10.1017/cts.2022.512

**Published:** 2022-12-14

**Authors:** Lori Carter-Edwards, Maia Hightower, Vanessa Schick, Tung Nguyen, Bertha Hidalgo, Lisa Cacari Stone, Rebecca Laird, Deborah Ossip, Mercedes Rubio, Sanae ElShourbagy Ferreira, Olveen Carrasquillo

**Affiliations:** 1 The North Carolina Translational and Clinical Sciences Institute (NC TraCS), University of North Carolina at Chapel Hill, Chapel Hill, NC, USA; 2 Kaiser Permanente Bernard J. Tyson School of Medicine, Pasadena, CA, USA; 3 Utah Clinical and Translational Science Institute, University of Utah, Salt Lake City, UT, USA; 4 Center for Clinical and Translational Sciences, University of Texas Health Science Center at Houston, Houston, TX, USA; 5 University of California San Francisco Clinical and Translational Science Institute, San Francisco, CA, USA; 6 Center for Clinical and Translational Science, The University of Alabama at Birmingham, Birmingham, AL, USA; 7 University of New Mexico Clinical and Translational Sciences Center, Albuquerque, NM, USA; 8 Center for Leading Innovation and Collaboration (CLIC), University of Rochester Clinical Translational Science Institute, Rochester, NY, USA; 9 Division of Training, Workforce Development, and Diversity, National Institute of General Medicical Sciences, National Institutes of Health, Bethesda, MD, USA; 10 Clinical and Translational Science Awards Program Branch, National Center for Advancing Translational Science, National Institutes of Health, Bethesda, MD, USA; 11 University of Miami Clinical and Translational Science Institute, Miami, FL, USA

**Keywords:** Clinical and Translational Science Award (CTSA), consortium, workforce, diversity, equity, inclusion, and accountability (DEIA), task force, accountability framework, learning system framework, recommendations

## Abstract

The Clinical and Translational Science Award (CTSA) Program recognizes that advancing diversity, equity, inclusion, and accessibility (DEIA) requires moving beyond statements of commitment to transformative actions. In 2021, the CTSA Program created a Task Force (TF) to initiate work in support of structural and transformational initiatives that advance DEIA for the consortium and its individual hubs. We describe the process of forming the expertise-driven (DEIA) TF and our activities to date. We 1) developed and adopted the DEIA Learning Systems Framework to guide our approach; 2) defined a set of recommendations across four focus areas (Institutional; Programmatic; Community-Centered; and Social, Cultural, Environmental); and 3) designed and disseminated a survey to capture the CTSA Program’s baseline demographic, community, infrastructural, and leadership diversity. The CTSA Consortium also elevated the TF to a standing Committee to extend our understanding, development, and implementation of DEIA approaches to translational and clinical science. These initial steps provide a foundation for collectively fostering environment that support DEIA across the research continuum.

## Background

The National Institutes of Health’s National Center for Advancing Translational Sciences (NIH, NCATS) highlights an environment promoting a culture to enhance workforce diversity as an essential characteristic of a successful Clinical and Translational Science Award (CTSA) Program [1,2]. This commitment is part of broader efforts to promote diversity and health equity across the NIH [3–6], the federal government [7], and throughout the medical and scientific communities [8,9]. In alignment with such efforts and with NCATS vision of bringing more treatments to all people more quickly [10], NCATS also prioritized ensuring clinical trials reflect population diversity [11,12].

Within the CTSA consortium, most Hubs recognize the importance of addressing diversity, equity, inclusion, and accessibility (DEIA). In a survey conducted at the CTSA Fall 2020 Program meeting (which focused on DEIA), an overwhelming majority (86%) reported being committed to making changes to improve DEIA. However, there was also recognition that despite considerable work in this area, only limited incremental progress had been made. As such, there was a call for the clinical and translational science (CTS) enterprise to move beyond statements of support to commitments toward transformative actions [13]. At the meeting, four goals and several strategies for achieving DEIA within the CTSA consortium were developed. The four goals emphasized transformational leadership, parity in funding for health equity and community-oriented research, community capacity building, and diverse enrollment for clinical trials [14]. Building on these efforts, on February 24, 2021, the CTSA Steering Committee announced that it planned to develop a DEIA Task Force (TF) to initiate the work needed to understand and advance structural DEIA efforts across the CTSA consortium. In this paper, we describe the strategic process of forming the TF, its activities, its operational framework, recommendations for structural actions, and development of an initial DEIA assessment tool.

## Methods

The CTSA Steering Committee sought to form the TF by inviting all CTSA Hubs [15] within the consortium to nominate up to two representatives with expertise in DEIA for consideration in the TF. For each candidate, Hubs were required to submit a NIH biosketch resume and a 150-word maximum statement of the candidate’s expertise and interest in DEIA. A total of 55 CTSA Hubs (87%) responded to the invitation. The CTSA Program Steering Committee reviewed candidates’ biographical pages and statements and selected 14 individuals to be members of the TF, creating a representative team across racial and ethnic groups, gender, geographic regions, and areas of expertise (Table 1). Those who were not selected received an email thanking them for their willingness to serve, informing them that they would be contacted if more opportunities to be engaged with the DEIA TF work arose. The CTSA Program Steering Committee cochairs also identified and appointed two cochairs to lead the TF (Table 1).

**Table 1.** Members of the CTSA diversity, equity, inclusion, and accessibility (DEIA) task force

Name	Institutional Affiliation	Role	State
Bruegl, Amanda	Oregon Health and Science University	Associate Professor Vice Chair of Diversity, Equity, and Inclusion Department of OB/Gyn Associate Director of the Education Core Northwest Native American Center of Excellence	Oregon
Cacari Stone, Lisa	University of New Mexico	Director, Transdisciplinary Research, Equity and Engagement Center	New Mexico
Carrasquillo, Olveen	University of Miami Medical Center	Professor of Medicine and Public Health Sciences Associate Dean for Clinical and Translational Research Co-Director of the Clinical and Translational Science Institute	Florida
Carter-Edwards^*^, Lori	University of North Carolina at Chapel Hill	Associate Professor, DEIA Faculty Liaison, North Carolina Translational and Clinical Sciences Institute	North Carolina
Coker, Robert “Trey”	University of Washington	Chair of the Steering Committee for the Northwest Participant Clinical Interactions Network	Washington
Haynes, Tiffany	University of Arkansas for Medical Sciences	Assistant Professor, Associate Director of the Community Engagement Core at the Translational Research Institute, Co-founder of Arkansas FAITH Network	Arkansas
Henderson, David	Boston University Medical Center	Psychiatrist-in-Chief, Professor and Chair of Department of Psychiatry and Co-Director of the Global Psychiatry Clinical Research Program	Massachusetts
Hidalgo, Bertha	University of Alabama at Birmingham	Associate Professor of Epidemiology, Center for Clinical and Transitional Science	Alabama
Hightower^*^, Maia	University of Utah	Chief Medical Information Officer, Associate Chief Medical Officer and Sr. Director of Health Equity Diversity, and Inclusion for the University of Utah Health	Utah
Lechuga, Claudia	Albert Einstein College of Medicine	Director of Research and Implementation Evaluation and Tracking	New York
Miller, Doriane	University of Chicago Medical Center	Associate Professor and Director of the Center for Community Health and Vitality, UChicago-Rush Clinical and Translational Science Award Hub’s Co-chair of the Community and Collaborations Cluster and Director of Health Equity Integration	Illinois
Nguyen, Tung	University of California San Francisco School of Medicine	Professor of Medicine, Leader of the CTSI Integrating Special Populations Core	California
Reid, Tisha	Purdue University	Senior Health Equity Analyst	Indiana
Schick, Vanessa	University of Texas Health Science Center at Houston	Associate Professor, School of Public Health	Texas
*CLIC and NCATS Team Members*			
Laird, Rebecca	University of Rochester Center for Leading Innovation and Collaboration (CLIC)	Assistant Director	
Ossip, Deborah	University of Rochester Center for Leading Innovation and Collaboration (CLIC)	Multi-Principal Investigator (MPI)	
Sanae ElShourbagy Ferreira	National Center for Advancing Translational Science (NCATS)		
Mercedes Rubio	National Center for Advancing Translational Science (NCATS) and National Institute of General Medical Sciences (NIGMS)		

* Diversity, Equity, Inclusion, and Acessability Task Force Co-Chair.

The charge to the TF was to increase CTSA Program consortium DEIA in clinical and translational research processes and workforce development. Also emphasized was the need to go beyond common, performative DEIA efforts in addressing DEIA to more structural, transformative initiatives. *Performative DEIA efforts* are statements or ritual practices declaring a commitment to DEIA but they avoid addressing well-entrenched, underlying cultural and institutional barriers or the policies, resources, and actions needed to promote DEIA [16]. While performative efforts may also include providing baseline data to inform change (e.g., reporting racial/ethnic demographics of the workforce), translating these performative efforts to actual change requires implementing specific action steps to address changes in power structures and decision-making within academic institutions to support DEIA. As a result, often such performative efforts simply maintain existing power and privilege structures. In contrast, *transformative DEIA efforts* are those that go beyond performative actions by fostering an equitable environment that engages in ongoing activities, policies, and initiatives that are inclusive, supportive, and promote growth for often marginalized or underrepresented populations [13].

At the outset, we developed three implementable short-term goals that could be accomplished during the 1-year work period related to this charge: a) develop a framework having a high-impact vision on diversity, inclusiveness, and health equity as an aim for the CTSA consortium at both a national and Hub-specific level; b) identify an initial set of recommendations for advancing DEIA within each Hub; and c) obtain a baseline understanding of where the CTSA Programs and Hubs are in addressing DEIA.

## Results

### The DEIA Learning System Framework (Fig. 1)

**Fig. 1.**
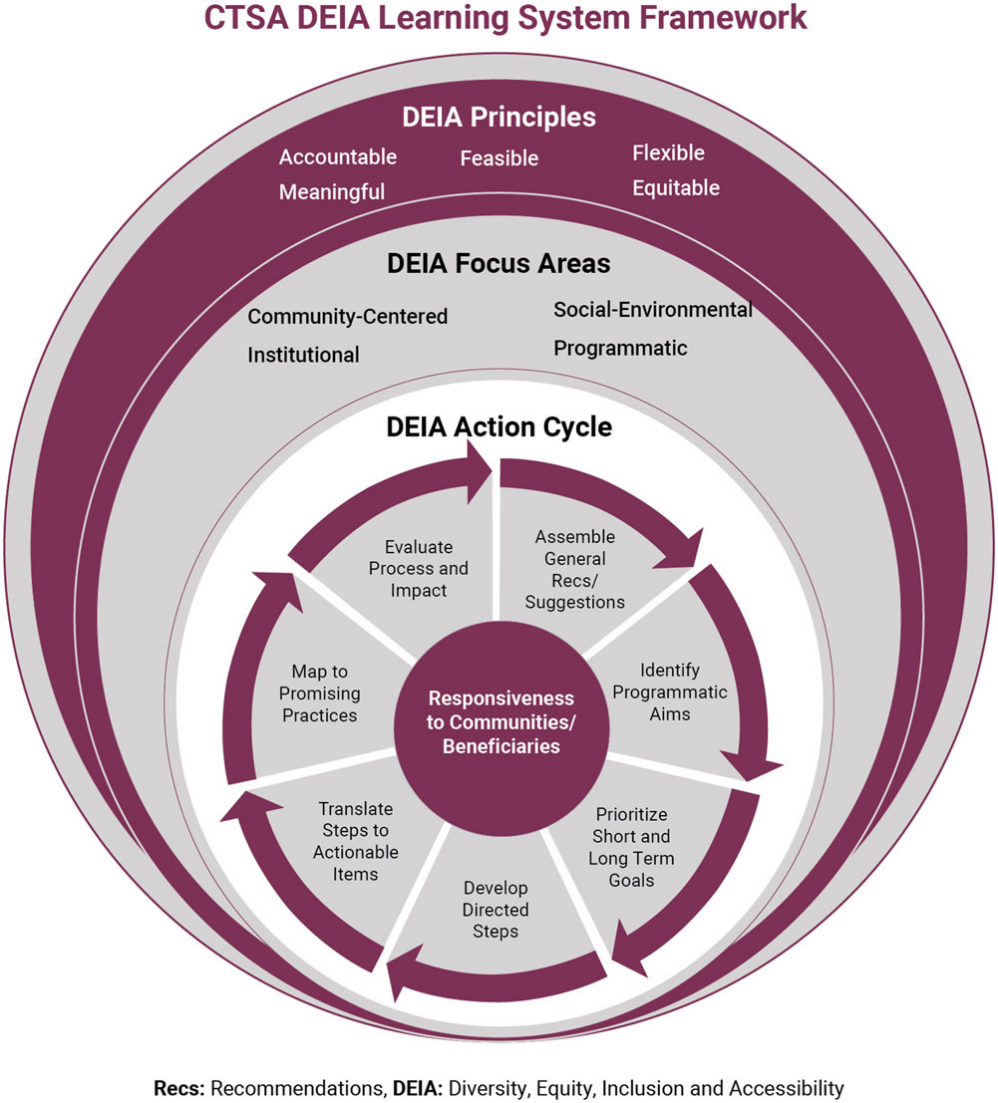
CTSA DEIA Learning Health System Framework.

Through a series of monthly meetings, emails, discussions, and phone calls, we organically developed a transformational DEIA framework (Table 2). Given the complexities of DEIA, and the fact that advancing DEIA has yet to be understood collectively in CTS, the initial step in developing the framework was agreement on five guiding principles to help drive DEIA efforts. These would allow the Hubs to be centered in a standard manner for gaining a greater understanding of DEIA and to normalize approaches to be shared across the consortium. These include:

1. Accountable – Collective learning within and across Hubs must take place to advance DEIA across the CTSA consortium. Reporting to and sharing regularly with diverse stakeholders is necessary to determine individual and collective progress.
2. Feasible – Addressing DEIA strategies and tactics that can be readily defined, conducted, measured, and reported by all of the CTSA Program Hubs within a short-time frame.
3. Flexible – The ability of the CTSA Program Hubs to adapt to infrastructural changes is necessary to institutionally promote DEIA that support improvement in translational science.
4. Meaningful – Conducting DEIA strategies and tactics that are deemed important to those traditionally underrepresented in the Academy and in local communities.
5. Equitable – Fostering an environment at the CTSA Program Hubs and within the CTSA Program consortium where voices and opinions from an expanded stakeholder community are heard when designing and implementing DEIA strategies and tactics.

**Table 2.** Glossary of diversity, equity, inclusion, and accessibility (DEIA)

*Diversity* involves recognizing and respecting different characteristics between individual and groups as assets when promoting health [17,18].
*Equity* is the state, quality or ideal of being just, impartial and fair, and health equity involves everyone having a fair opportunity to attain their full health potential and that no one should be disadvantaged from achieving this potential [19].
*Inclusion* is the establishment and maintenance of a culture that is collaborative, respectful, and supportive, embracing differences and respecting individuals and groups in actions as well as words [17].
*Accessibility* involves providing equal access and opportunity to as many people as possible. This may include, for example, resources (such as digital access), learning opportunities, and career opportunities [20].

The next step in developing the framework was agreement on focus areas for the CTSA consortium, which would drive recommendations for action. Based on the goals and strategies from the Fall 2020 CTSA meeting [14], the TF developed the following four DEIA focus areas:

1. Institutional – The CTSA Program, Hub-specific institutional policies, and within-institutional relationships.
2. Programmatic – Hub-specific training programs and initiatives.
3. Community-Centered – Interrelatedness of community engagement and efforts to end health disparities in communities of greatest need.
4. Social/Cultural/Environmental – Work environment and social spaces and situations where biases and microaggressions are embedded in day-to-day activities.

Guided by these principles and focus areas and also inspired by learning health system frameworks [8–11], the TF then developed the DEIA Action Cycle. This iterative component of the framework has seven stages aimed at achieving DEIA progress. The first stage is *general recommendations and suggestions* for addressing DEIA generated within the CTSA Hub in whatever format is deemed suitable by each Hub. The second stage is *identifying objectives* that the Hub intends to implement to advance its work. These should be SMART objectives (Specific, Measurable, Achievable, Relevant, Time-bound) [21,22] and may be at multiple levels within the Hub, as well as influenced by institutional policies and procedures. Hubs should then *prioritize* parallel approaches balancing both *short- versus long-term objectives.* This entails critically reviewing what can be accomplished to demonstrate immediate progress alongside broader priorities that may require more complex infrastructural efforts. This balance is essential as focusing only on long-term priorities may miss the identification of the smaller wins through which Hubs can demonstrate rapid progress. Conversely, only focusing on short-term objectives may leave out critical longer-term strategies addressing complex infrastructural barriers.

Each Hub would then develop *directed steps* to meet its objectives. These steps would then be *translated into actionable items* including activities that can lead to recognizable outputs. Such items should also be *mapped to promising practices*, which eventually would be replicated and shared with other Hubs, individually and collectively. *Evaluation* involves assessing whether the goals and objectives were met, whether there were challenges, and identifying the lessons learned for the next iteration of recommendations and suggestions associated with the prior work conducted, thereby repeating the accountability cycle, and building on DEIA successes to create a sustainability culture. Central to each stage of the DEIA Action Cycle is that all steps need to be continuously responsive to communities and beneficiaries with input and consultation with such stakeholders at each step of the process. Whether through communication strategies, engagement, or infrastructure modifications, ensuring all activities are responsive to these vitally important stakeholders is a fundamental element of the framework.

The product of our efforts was the DEIA Learning System Framework which the TF proposes as a guide for the development of the recommendations and proposed actions by the Hubs and the consortium (Fig. 1). Regardless of the phase or readiness of the CTSA, each Hub can adopt this framework to be used at any level, whether with leadership or staff, or within the CTSA Hub and larger institution. Additionally, this DEIA learning system framework can be applicable, not only among CTSA Hubs, but also broadly across many different programs and centers.

### DEIA Recommendations for CTSAs

Along with development of the framework, we also sought to identify an initial set of DEIA recommendations for each of the four focus areas. In August 2021, TF members were assigned to one of four ad hoc groups (each with 3–4 members). Each group was tasked with developing focus area-specific recommendations to help Hubs shift from performative to transformative approaches for advancing DEIA, in partnership with their affiliate institutions. To guide the small-group discussions, each ad hoc group referred to a set of four facilitating questions: 1) What focus area “ground truths” will be used to help build our DEIA framework? 2) What major concerns exist (if any) after review of the Fall 2020 CTSA Program summary report? 3) What may be missing from the recommendations?, and 4) What can be considered short- or long-term goals?

The initial recommendations by the ad hoc groups were reported to the larger TF and subsequently refined through additional input at monthly meetings and through online communications. Table 3 displays the recommendations for each of the four focus areas, including their descriptions and examples of activities for implementation. The *institutional* focus area includes recommendations that emphasize creating infrastructural strategies that promote meaningful opportunities for aligning DEIA program goals and leadership workforce development. The *programmatic* recommendations also address infrastructural change, with emphasis on DEIA capacity building through training environments for researchers and communities and more diverse, inclusive research teams.

**Table 3.** Diversity, equity, inclusion, and acessability task force recommendations by focus area

Focus Area	Recommendation	Description	Example(s)
Institutional	1. Develop broad institutional commitment to Diversity, Equity, Inclusion, and Acessability	Build partnerships by actively collaborating with departmental, institutional, and across-institutional leadership on common/aligned programmatic goals to catalyze change in climate	• Implement an intentional meeting cadence between multiple levels of institutional leadership to ubiquitously prioritize Diversity, Equity, Inclusion, and Acessability. • Create opportunities for employees to share ideas and strategies that can be considered at the institutional level.
	2. Develop Leader and Leadership Pathways	Make room for structural change in Diversity, Equity, Inclusion, and Acessability efforts by sharing space, influence, and resources – including leadership positions with commensurate salary –with scientists who have demonstrated their commitment to such work	• Find ways to recognize the broad, institutional value these scientists bring to this field, which may help: • Improve the quality of research conducted with and within populations of greatest impact. • Increase the institution’s Diversity, Equity, Inclusion, and Acessability scientific workforce.
Programmatic	1. Develop Diversity, Equity, Inclusion, and Acessability Conducive Training Environments	Support learning communities to share effective tools, methods, and findings, and the stories that show their use, both within and across local communities, and within and across CTSAs. Also require and provide training in the principles and methods of community engagement for all who engage in research with community members and organizations.	• Provide infrastructural support to share tools, methods, findings, and exemplar stories among learning communities. • For training in the principles and methods of community engagement: • Require it for all who engage in research with community members and organizations. • Offer it to all who engage in research. • Provide opportunities for ongoing consultation with the community.
	2. Build Diversity, Equity, Inclusion, and Acessability into Research Teams	Engage in a series of coordinated steps to educate and raise awareness, and to take specific actions to create diverse teams, foster community partnerships, and support community-engaged research.	• Train research teams in cultural humility and effective communications. • Embed researchers with expertise in minority health and health disparities into research teams. • Hire individuals with experience working in communities. • Provide opportunities for training in research to interested community members. • Build capacity for community members to engage in research. • Provide opportunities for training in research to interested community members. • Provide no-cost research consultations to community members engaged in research.
Community-Centered	1. Support research for the community by understanding area demographics	Know the demographic composition of your primary service area in comparison to demographics of clinical trial participants.	• Provide support for tools and data infrastructure to track the diversity of clinical trial participants across priority populations. • Implement system-wide processes for monitoring participants of clinical trials and produce periodic reports on the demographic representation of trial participants • Require oversampling of underrepresented populations in clinical trials. This is especially important in disease states where marginalized populations are disproportionately affected. • Increase the inclusion of underrepresented groups by combining datasets across Clinical and Translational Science Awards and partnering institutions and facilitate the use of mixed methods of inquiry in accessing such datasets
	2. Support research for the community	Formally assess the community needs and priorities.	• Access institutional and community health data available across the Hub research enterprise to encourage alignment between research and community priorities (one example may be the Community Health Needs Assessments [CHNA] which includes input from community leaders reflecting the diversity of the communities they serve).
	3. Engage in research with the community	Increase the: a) number of quality relationships and b) level of engagement with community members.	• Utilize existing frameworks for community engagement to guide activities. (e.g., Patient Centered Outcomes Research Institute, [23] or National Academy of Medicine [24]) • Include quantitative metrics that measure the quality of the engagement. • Acknowledge and identify gaps of populations where relationships may not exist and need to be forged (e.g., indigenous populations in the region and recognize that American Indian and Alaska Native (AI/AN)and other vulnerable populations which may be geographically dispersed in both urban and rural areas).
Social, cultural, environmental	1. Identify and dismantle assumptions	Dismantle workforce assumptions of the top-down approach as the primary method for recognizing the strengths and contributions of multidisciplinary teams and multipartnered research.	• Recognize the value and expertise of the entire health profession including the community partners, Minority Serving Institutions, and project staff (not just senior faculty or MDs),
	2. Amplify diverse voices across the translational science continuum	Bring community and staff voices within the translational science continuum by increasing decision-making authority and by bolstering workforce development.	• Increase multiple principle investagator mechanisms that include community partners. • Link community voices to all stages of the translational continuum including Tier 1 basic science initiatives, creating bi-directional opportunities for learning. • Expand dissemination protocols beyond publication (built-in preliminary findings feedback loops). • All research studies must have a plan on how they will share finding with community partners as well as research peers.

The *community-centered* recommendations, like the programmatic ones, emphasize engagement with and support for communities. There is recognition of importance of study data that are representative of the area and are inclusive of community input and demographics in conducting research. For this recommendation, CTSA Hubs are encouraged to self-define a population-based geographic area that they primarily serve. While all CTSAs are engaged in research of broad applicability and impact, this geographic area is the one each Hub intends to primarily serve with respect to the communities it engages, the outreach it performs, and research it conducts.

The *social, cultural, and environmental* recommendations center on actionable ways to demonstrate the value of a diverse team of researchers, staff, community members, and partnering organizations when conducting work along the research continuum. These recommendations align with those in the institutional focus area. Examples for all of these recommendations are initial ways to operationalize efforts to improve structural approaches as well as human interaction that is diverse, equitable, and inclusive.

It is also understood that each CTSA is unique in its demographic service area, mission, operations, and needs. These factors will influence how each CTSA prioritizes and implements these recommendations. In addressing these focus areas, Hubs will have different DEIA capacities and levels of readiness unique to their CTSA environment. For instance, one Hub may prioritize building its continued capacity in community-centered DEIA, whereas another may seek to target institutional DEIA efforts, and another may choose to concentrate on social-environmental and programmatic DEIA activities. This will be determined by the CTSA Hub, its parent institution, its affiliate institutions (where applicable), and its partnering community organizations. However, it is the hope that all Hubs aspire to improve DEIA in various areas and share with the consortium promising practices, challenges, and lessons learned. By doing so, we can improve our infrastructures and foster more constructive environments to collectively improve and advance CTS across the research continuum.

### DEIA Survey

Our third goal was to conduct an initial DEIA assessment designed to help us identify a baseline understanding of *diversity* across the CTSA Program consortium and within individual Hubs and affiliated partners and collaborators. Through an iterative process among TF members, we developed a diversity survey that each Hub is being asked to complete. If there are multiple institutions within the Hub, they can provide supplemental reports. The survey includes questions to assess: 1) demographic characteristics of the community served; 2) demographic diversity (e.g., race, ethnicity, gender, disability, age) of CTSA Program Hub leadership and staff; 3) characteristics of community engagement staff and activities; 4) infrastructure to support DEIA efforts (e.g., diversity/health equity office); 5) perceived representativeness of Hub staff and leadership relative to community; and 6) existence and use of a DEIA dashboard. CTSA Hubs will be asked to save their individual-level responses for their own internal review and understanding. Individual-level data will be de-identified and only disseminated at an aggregate level and cannot be used to compare Hubs. The hope is to ultimately develop a longitudinal series of assessments to help us better understand the state of DEIA across the CTSA Hubs.

### Presentations and the TF’s 1-Year Progress

The framework and recommendations were shared with the CTSA Program National Steering Committee on November 30, 2021, and May 9, 2022. Information was also disseminated to the CTSA consortium through the CTSA listservs, in the monthly consortium newsletter (“The Ansible”), through a presentation by the TF at the CTSA consortium monthly Program Webinar, and through a presentation to the Evaluation Special Interest Group on April 19, 2022, prior to the Association for Clinical and Translational Science annual meeting. On April 18, 2022, the CTSA Steering Committee elevated the DEIA from a time-limited TF to an Enterprise Committee (EC). The CTSA ECs are long-term committees that function to advance the field of translational science through ongoing open discussions, project planning, and metric assessment. The DEIA is the fifth EC of the CTSA Program. The DEIA survey was also presented to the CTSA Program Steering Committee and modified based on their feedback. It was then approved for dissemination by the CTSA Program Steering Committee and distributed to hubs on July 8, 2022. Findings of the survey will be presented at the Fall 2022 CTSA Program meeting in November 2022, and subsequently disseminated for broad use.

### Summary

There is increased national recognition that our health care and public health systems must change structurally if we are serious and intentional about turning the tide on historical, disparate health outcomes [25]. The COVID-19 pandemic revealed that this reckoning must include the transformation of healthcare’s institutional infrastructures, including care delivery, education, and research, that help perpetuate systemic health inequities. These include, but are not limited to, those experienced due to race, place, ethnicity, and gender [26–29]. Central to achieving this goal are transformative efforts in support of DEIA. In this report, the CTSA DEIA EC puts forward a framework and set of recommendations for the CTSA consortium to collectively achieve our common goal of addressing DEIA. Recognizing that each Hub has unique characteristics and needs, to promote CTS that has a sustainable impact in DEIA, CTSA Hubs need to self-identify and address their own unique capacities. Also important is collaborating with the CTSA consortium to share and learn from promising practices to take collective, constructive action to improve DEIA and collectively improve how we conduct CTS. The survey findings will further inform DEIA efforts across the consortium and beyond.
